# A novel iterative detection method based on a lattice reduction-aided algorithm for MIMO OFDM systems

**DOI:** 10.1038/s41598-024-52602-6

**Published:** 2024-02-02

**Authors:** Haitao Liu, Xuchao Cheng, Wenqing Li, Fan Feng, Liguo Wang, Ying Xiao, Shiqi Fu

**Affiliations:** 1 College of Information and Communication Engineering, Dalian Minzu University, Dalian, China; 2 School of Software Technology, Dalian University of Technology, Dalian, China; 3 KEDE Numerical Control Co, LTD, Dalian, China

**Keywords:** Electrical and electronic engineering, Information technology

## Abstract

The lattice reduction-aided algorithm has received broad attention from researchers since it operates as a maximum likelihood receiver with better system performance for multiple-input multiple-output orthogonal frequency division multiplexing systems and contains a full diversity. A novel iterative detection algorithm canceling parallel iterations that employ the lattice reduction-aided approach is proposed. Soft information is exchanged through the detector itself. Its iteration occurs inside the detector, which reduces much of the exchange cost between the multiple-input multiple-output orthogonal frequency division multiplexing detector and the turbo decoder. Since the parallel interference cancellation algorithm is constrained by the accuracy of the initial value of the detection, it is easy to form error propagation after several iterations. Due to the lattice reduction-aided algorithm, its performance is approximated with the maximum likelihood algorithm. Therefore, the lattice reduction-aided algorithm is introduced into the parallel interference cancellation algorithm to make its detection algorithm more accurate and overcome the effect of error propagation in the manuscript. Simulation results indicate that the proposed algorithm leads to an improvement of 0.8–2 dB when the bit error rate is set to 10^–4^ when compared to other algorithms.

## Introduction

Communication systems employing Multiple Input Multiple Output Orthogonal Frequency Division Multiplexing (MIMO-OFDM) play a crucial role in current and future implementations and have garnered w idespread attention from researchers^1–3^. Compared to single-antenna systems, MIMO systems offer diversity gains, enabling higher transmission rates, reliability, and a more consistent channel. One of the primary advantages of MIMO is transmitting distinct information streams from different transmitting antennas, also known as spatial multiplexing. However, reliable detection of these information streams at the receiving end is essential to ensure data accuracy.

In order to achieve the optimal bit error rate, the maximum likelihood (ML) algorithm has the best performance, considering all propagation vectors. However, as the number of transmitting antennas increases, the algorithm's complexity grows exponentially, generally not considered alone. Detection algorithms mainly consist of straightforward methodologies, where Sphere Decoding (SD) is a well-known detector, but it is practically limited to 32 dimensions. Therefore, linear detection algorithms represented by Zero Forcing (ZF)^4^ and Minimum Mean Square Error (MMSE)^5^ are proposed. The ZF detector ignores the noise interference of the channel, leading to poor performance as it amplifies noise while eliminating interference. The MMSE algorithm strikes a balance between interference elimination and noise amplification, generally outperforming the ZF detection algorithm. Although both ZF and MMSE algorithms can achieve low-cost signal detection, their detection performance is not high^6^.

To enhance the performance of the algorithm, researchers have proposed non-linear detection algorithms^7^. Successive Interference Cancellation (SIC) is a representative non-linear detection algorithm, composed of layer-by-layer techniques to enhance the signal, depending on the initial score of the first detection^8^. However, it is more susceptible to Error Propagation (EP). Linear Receivers Aid (LRA) serves as a highly effective preprocessing method, typically combined with other techniques^9^. An MIMO-OFDM receiver supporting LRA begins by recognizing a set of small matrices that are nearly orthogonal to the specified channel matrix. In algorithms employing LRA^10,11^, if a lattice is given, it can generate an almost orthogonal matrix. A QR decomposition-based LLL algorithm is proposed in literature^12^. The literature^13^ proposes an SIC detection algorithm assisted by an LLL algorithm, and although the use of the LLL-assisted algorithm improves the performance of the SIC detection algorithm, error propagation may still occur.Preprocessing the channel matrix optimizes its dimensions and reduces inter-vector correlation. In literature^14,15^, these methods achieve outstanding performance by lowering complexity. Although its performance advantage over iterative detection algorithms is considerably smaller, its simplicity is more straightforward compared to iterative methods. Therefore, using simple iterative algorithms at a certain complexity is highly suitable. However, the detection algorithm based on QR decomposition essentially constitutes a form of SIC detection, involving error propagation. Therefore, literature^16^ proposes a Sorted QR Decomposition (SQRD) detection algorithm. The algorithm uses a modified Gram-Schmidt orthogonalization algorithm to sort the channel matrix, prioritizing the detection of better-performing layer signals, thus suppressing error propagation. However, this algorithm does not effectively address the problem of low diversity in the initially detected layers. In reference^17^, a Modified MMSE-SQRD detection algorithm is proposed. By moving this symbol component into the signal term, the modified MMSE SQRD based detection can provide more efficient LLRs for soft decoder. Literature^18^ proposes an Iterative QR Decomposition (IQRD) detection algorithm. The algorithm utilizes iterative cycles, retaining only the signals from the last detection layer in each iteration process. This approach addresses the issue of low diversity in the initially detected layers.

For joint decoding communication systems, the Maximum A Posteriori (MAP) criterion stands as one of the representative optimal detection methods. However, due to the high complexity of the MAP algorithm, its implementation is limited in many practical applications. In literature^19^, low-complexity SIC and MMSE-based multi-user detectors are proposed for multipath CDMA channels. Subsequently, similar SIC-MMSE detectors are applied in iterative reception algorithms^20^. However, the complexity of such SIC methods remains relatively high. Therefore, researchers have proposed a novel, low-complexity nonlinear receiver algorithm that parallelly decodes data streams through zeroing and elimination. Parallel Interference Cancellation (PIC) is a well-known method. The PIC algorithm can effectively reduce the algorithm's complexity and improve its performance through iteration. These iterative algorithms^16,21–23^ represent the best detection methods in suboptimal algorithms, excluding the optimal MAP algorithm. However, all these algorithms exchange information through mutual MIMO detectors and decoders. Although it brings performance improvement, it also consumes a significant amount of time and is relatively complex.

The exchange of information between detectors and decoders to achieve signal detection introduces a significant amount of additional computation. This paper proposes an Iterative Parallel Interference Cancellation algorithm based on lattice reduction-aided (IPIC-LRA), where detectors themselves provide mutual feedback and iteration. Since the Precision of Initial Detection Values is crucial for the PIC algorithm, if the first detection process fails, the initial value's detection is not ideal. Consequently, it leads to error propagation, severely impacting the algorithm's performance.

The proposed IPIC-LRA algorithm in this paper combines the non-joint detection and decoding of the PIC and lattice reduction algorithms. It separates the detection in the first iteration from subsequent iterations, enhancing detection accuracy. Additionally, it employs a lattice reduction auxiliary algorithm for assistance. Simulation results demonstrate that, compared to the original algorithm at the same bit error rate, the IPIC-LRA algorithm with the lattice reduction exhibits a significant improvement in signal-to-noise ratio. Moreover, due to separate computations, the algorithm's complexity remains almost unchanged.

The rest of the article is outlined as follows: The system model is described in Section “The model of the system”. Section “Iterative parallel interference cancellation algorithm based on lattice reduction-aided (IPIC-LRA) approach” describes an iterative parallel interference cancellation algorithm based on a lattice reduction-aided approach (IPIC-LRA) in detail. The complexity of the proposed method is discussed in Section “Complexity”. Section “The results of the simulation” presents simulation steps and results. The paper is concluded in Section “Conclusion”.

## The model of the system

To achieve soft detection, the joint decoding of the MIMO-OFDM wireless systems with $N_{T}$ transmits and $N_{R}$ receiving antennas should be taken into consideration. This approach could lead to increased efficiency and robustness in wireless communications. The combination of the two techniques, MIMO and OFDM, could provide significant improvements in terms of data transmission accuracy and channel capacity. When data going through the encoder output is denoted $c\left( n \right)$, it can then be obtained by random interleaving. Finally, the modulated signal is obtained after running modulation. The 4QAM modulation schemes are utilized throughout the paper. Then, the final transmitted signal $x_{{N_{t} }} \left( k \right)$ is obtained. The transmission signal vector ${\mathbf{X}}_{{\text{c}}} \in {\mathbb{C}}^{{N_{T} \times 1}}$,${\mathbf{X}}{ = }\left( {x_{{N_{1} }} ,x_{{N_{2} }} \cdots x_{{N_{T} }} } \right)^{{\text{T}}}$, with energy *ES* is expressed by ${\mathbf{R}}_{{{\mathbf{xx}}}} = E\left[ {{\mathbf{xx}}^{H} } \right] = {\mathbf{I}}$, where ${\mathbb{C}}$ represents the signal set. ${\mathbb{C}} = \left\{ { \pm \frac{a}{2}, \pm \frac{3a}{2}, \ldots , \pm \frac{\sqrt M - 1}{2}a} \right\} + j\left\{ { \pm \frac{a}{2}, \pm \frac{3a}{2}, \ldots , \pm \frac{\sqrt M - 1}{2}a} \right\}$, $a = \sqrt {\frac{6}{M - 1}}$. $a$ represents the energy normalization coefficient, and $M$ denotes the modulation order. A received signal vector ${\mathbf{Y}}{ = }\left( {{\text{y}}_{{N_{1} }} {,}y_{{N_{2} }} {,} \cdots {,}y_{{N_{R} }} } \right)^{{\text{T}}}$ is presented as follows.

1 $$ {\mathbf{Y}} = {\mathbf{HX}} + {\mathbf{N}}{ = }\sum\limits_{i = 1}^{{N_{R} }} {{\mathbf{h}}_{i} {\mathbf{x}}_{i} + } {\mathbf{n}} $$

where the complex noise vector ${\mathbf{N}} \in N_{R} \times 1$ can be represented by ${\mathbf{N}}{ = }\left( {n_{{N_{1} }} {,}n_{{N_{2} }} {,} \cdots {,}n_{{N_{R} }} } \right)^{{\text{T}}}$, and ${\mathbf{R}}_{{{\mathbf{nn}}}} = E\left[ {{\mathbf{nn}}^{H} } \right] = \sigma^{2} {\mathbf{I}}$,$\sigma^{2}$ represents the variance of the complex noise vector. When the ideal channel estimation condition is assumed, the channel matrix ${\mathbf{H}} \in N_{R} \times N_{T}$ in Eq. (1) can be given by the ${\mathbf{H}}$ matrix.

2 $$ {\mathbf{H}} = \left[ {\begin{array}{*{20}l} {{\mathbf{h}}_{1,1} } \hfill & {{\mathbf{h}}_{{1,2}} } \hfill & \cdots \hfill & {{\mathbf{h}}_{{{1,}N_{T} }} } \hfill \\ {{\mathbf{h}}_{2,1} } \hfill & {{\mathbf{h}}_{{2,2}} } \hfill & \cdots \hfill & {{\mathbf{h}}_{{{2,}N_{T} }} } \hfill \\ \vdots \hfill & \vdots \hfill & \ddots \hfill & \vdots \hfill \\ {{\mathbf{h}}_{{N_{R} ,1}} } \hfill & {{\mathbf{h}}_{{N_{R} {,2}}} } \hfill & \cdots \hfill & {{\mathbf{h}}_{{N_{R} {,}N_{T} }} } \hfill \\ \end{array} } \right] $$

where ${\mathbf{h}}_{j,i} \in \Re^{2 \times 2}$ can be expressed by

3 $$ {\mathbf{h}}_{j,i} = \left( {\begin{array}{*{20}l} {\Re \left[ {h\left( {j,i} \right)} \right]} \hfill & { - \Im \left[ {h\left( {j,i} \right)} \right]} \hfill \\ {\Im \left[ {h\left( {j,i} \right)} \right]} \hfill & {\Re \left[ {h\left( {j,i} \right)} \right]} \hfill \\ \end{array} } \right) $$

where ${\mathbf{h}}_{j,i}$ represents the channel impulse response between the jth receiving antenna and the ith transmitting antenna. At the receiver, the signals $y_{{N_{r} }} \left( k \right)$ are demodulated by the OFDM symbol, the soft detection input value then can be obtained. The goal of the algorithm is to generate the LLR information corresponding to the emission vector ${\mathbf{x}}\left( k \right)$ with the ${\mathbf{h}}\left( k \right)$ and ${\mathbf{y}}\left( k \right)$ of each coded bit. The maximum a posteriori probability (MAP) algorithm has a higher complexity when the likelihood ratio information is computed. To calculate the estimated signal value ${\widehat{x}}_{i}(k)$, it is appropriate to employ linear detection to calculate the LLR. Then, the likelihood ratio information of each symbol is computed according to the mean and the variance of each symbol's estimated score to decrease the complexity.

## Iterative parallel interference cancellation algorithm based on lattice reduction-aided (IPIC-LRA) approach

### The channel matrix reconstruction based on the LLL and equivalent change of the received signal

According to Eq. (1), the MIMO-OFDM system could be regarded as a lattice structure on a complex field. The basis vector with column vectors as a lattice is formed by

4 $$ L({\mathbf{\rm H}}) = L\left( {h_{1} ,h_{2} , \ldots ,h_{{N_{t} }} } \right) = \sum\limits_{i = 1}^{{N_{t} }} {h_{i} x_{i} } $$

The necessary condition for ${\mathbf{\overline{\rm H}}} = {\mathbf{{\rm H}T}}$ generating the same lattice ${\mathbf{\rm H}}$ is that **T** must be a unimodular matrix^24^. Lattice reduction technology is implemented to optimize the channel matrix so that the optimized base vector converted into a shorter vector with better orthogonality could be a better decision domain. While the LLL algorithm holds the title of the most frequently employed lattice reduction technique, the complex LLL algorithm garners increasing interest as it offers lower computational complexity than the LLL.

The main reason for the bad performance of the linear detection algorithm is that the high orthogonality of the channel matrix is destroyed. The MIMO-OFDM detection method used on almost orthogonal matrices could improve performance. Therefore, the LLL algorithm deserves more attention. Thus, the channel matrix is expanded to:

5 $$ {\overline{\mathbf{H}}} = \left[ \begin{gathered} {\mathbf{H}} \hfill \\ \sigma_{n} {\mathbf{I}}_{{N_{T} }} \hfill \\ \end{gathered} \right] $$

where ${\overline{\mathbf{H}}} \in {\mathbb{R}}^{{2\left( {N_{T} + N_{R} } \right) \times 2N_{T} }}$ and ${\mathbf{I}}_{{N_{T} }} \in {\mathbb{R}}^{{2N_{T} \times 2N_{R} }}$ represent the extended channel matrix and the identity matrix. In Eq. (4), ${\mathbf{H}}$ and ${\overline{\mathbf{H}}}$ are of the size $2N_{T} \times 2N_{R}$ and $2\left( {N_{T} + N_{R} } \right) \times 2N_{T}$, respectively.

If the channel is extended, the received signal needs to be processed accordingly with zero padding, and the extended signal ${\overline{\mathbf{Y}}} \in \Re^{{2\left( {N_{T} + N_{R} } \right) \times 1}}$ is determined by

6 $$ {\overline{\mathbf{Y}}} = \left( \begin{gathered} {\mathbf{Y}} \hfill \\ {\mathbf{0}}_{{2N_{T} }} \hfill \\ \end{gathered} \right) = {\overline{\mathbf{H}}\mathbf{X}} + \left( \begin{gathered} {\mathbf{N}} \hfill \\ - \sigma_{n} {\mathbf{X}} \hfill \\ \end{gathered} \right) $$

where ${\mathbf{0}}_{{2N_{T} }}$ denote the $2N_{T}$ dimensional null vector. The first part to the right-hand side of Eq. (5) is the signal vector, and the second part is a composite form of both the noise and transmission signal vectors. If the second part is treated as noise, the detection performance will be affected.

The variance $\sigma_{n}$ and unit matrix ${\mathbf{I}}_{{2N_{T} }}$ are utilized to fill the ${\mathbf{H}}$. Then QR decomposition is denoted as follows:

7 $$ {\overline{\mathbf{H}}} = \left[ \begin{gathered} {\mathbf{H}} \hfill \\ \sigma_{n} {\mathbf{I}}_{{2N_{T} }} \hfill \\ \end{gathered} \right]{ = }{\mathbf{\overline{Q}\overline{R}}} = \left[ \begin{gathered} {\mathbf{Q}}_{1} \hfill \\ {\mathbf{Q}}_{2} \hfill \\ \end{gathered} \right]{\overline{\mathbf{R}}} $$

After conducting both the channel matrix expansion and QR decomposition, ${\overline{\mathbf{Q}}}$ and ${\overline{\mathbf{R}}}$ is introduced into the LLL algorithm to reconstruct the channel matrix ${\mathbf{H}}_{{{\text{equ}}}}$. Thus, Eq. (5) can be rewritten as

8 $$ {\overline{\mathbf{Y}}} = {\mathbf{H}}_{{{\text{equ}}}} {\mathbf{T}}^{ - 1} {\mathbf{P}}^{ - 1} {\mathbf{X}} + {\overline{\mathbf{N}}} $$

The matrix ${\mathbf{P}}$ is the sequence order of the detected symbols after the decomposition of the channel ${\mathbf{H}}$ by SQRD, which represents a column arrangement. To further clarify the expression, the matrix ${\mathbf{M}} = {\mathbf{H}}_{{{\text{equ}}}} {\mathbf{T}}^{ - 1} {\mathbf{P}}^{ - 1}$ is written.

### The analysis of the algorithm

**Figure 1:**
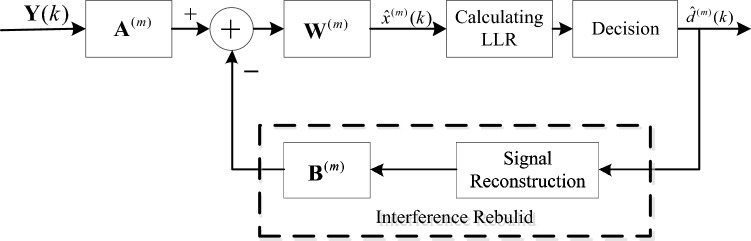
A block diagram of the IPIC-LRA.

Figure 1 depicts the block diagram of the IPIC-LRA. The received signal on the antenna at the receiver side is obtained after conducting de-CP, IFFT transform, and de-carrier mapping. So, the input signal ${\mathbf{Y}}\left( k \right)$ of the algorithm is obtained. The matrix ${\mathbf{A}}^{\left( m \right)}$ represents the forward matrix, the $m$ denotes the number of different iterations. The form of the forward matrix changes based on different $m$ values. The matrix ${\mathbf{B}}^{\left( m \right)}$ represents the feedback matrix, which is used to reconstruct the antenna interference. The matrix ${\mathbf{W}}^{\left( m \right)}$ denotes the normalized matrix, which is used to normalize the received signal power. According to Fig. 1, the estimated value of the transmitted signal could be attained after running the $m$-th iteration and is denoted by

9 $$ \hat{x}^{\left( m \right)} \left( k \right) = {\mathbf{W}}^{\left( m \right)} \left[ {{\mathbf{A}}^{\left( m \right)} {\mathbf{Y}}(k) - {\mathbf{B}}^{\left( m \right)} \hat{x}^{{\left( {m - 1} \right)}} \left( k \right)} \right] $$

The first detection method of the IPIC algorithm based on a lattice reduction-aided approach is different from the subsequent detection. The principle will be explained in detail in the next section.

- The first iteration ($m = 1$)

The same detection algorithm is applied to all the subcarriers for the MIMO-OFDM systems, so the analysis of the algorithm in the manuscript starts by considering the case of the $k$ carriers. Since there is no feedback a priori information during the first iteration the detection of the received signal at this time is assumed to be linear. According to the conventional PIC detection algorithm in the first iteration, the linear MMSE detection algorithm is chosen, and the forward matrix ${\mathbf{A}}^{(1)} \left( k \right)$ is defined by

10 $$ {\mathbf{A}}^{(1)} \left( k \right) = \left( {{\mathbf{H}}_{{{\text{equ}}}}^{H} \left( k \right){\mathbf{H}}_{{{\text{equ}}}} \left( k \right)} \right)^{ - 1} {\mathbf{H}}_{{{\text{equ}}}}^{H} \left( k \right) $$

When the case of multiple different subcarriers is considered, the forward matrix ${\mathbf{A}}^{(1)}$ is a block diagonal matrix composed of different subcarrier forward matricesfankui denoted by

11 $$ {\mathbf{A}}^{(1)} = \left[ {\begin{array}{*{20}l} {{\mathbf{A}}^{(1)} \left( 1 \right)} \hfill & 0 \hfill & \cdots \hfill & 0 \hfill \\ 0 \hfill & {{\mathbf{A}}^{(1)} \left( 2 \right)} \hfill & \cdots \hfill & 0 \hfill \\ \vdots \hfill & \vdots \hfill & \ddots \hfill & \vdots \hfill \\ 0 \hfill & 0 \hfill & \cdots \hfill & {{\mathbf{A}}^{(1)} \left( N \right)} \hfill \\ \end{array} } \right]_{{2N_{T} N \times 2N_{R} N}} $$

where $N$ denotes the number of data carriers. Since there is no feedback signal during the first detection, there is no need to reconstruct the interference signal, then the feedback matrix ${\mathbf{B}}^{(1)} \left( k \right)$ at this time is defined by

12 $$ {\mathbf{B}}^{(1)} \left( k \right) = 0 $$

There is no interference-canceling process in the operation, so, no power normalization is required, and the matrix ${\mathbf{W}}^{(1)} \left( k \right)$ is defined by

13 $$ {\mathbf{W}}^{(1)} \left( k \right) = {\mathbf{I}}_{{2N_{T} N}} $$

Both the feedback and the normalization matrices, ${\mathbf{B}}^{(1)}$ and ${\mathbf{W}}^{(1)}$, satisfy the block diagonal matrix consisting of ${\mathbf{B}}^{(1)} \left( k \right)$ and ${\mathbf{W}}^{(1)} \left( k \right)$. The signal estimates for the first iteration are obtained by employing Eqs. (10) through (13) and lead to

14 $$ \begin{gathered} {\hat{\mathbf{d}}}^{1} = {\mathbf{A}}^{(1)} {\overline{\mathbf{Y}}} \\ \, = {\mathbf{\Phi d}} + {\mathbf{A}}^{(1)} {\mathbf{n}} \\ \end{gathered} $$

where ${{\varvec{\Phi}}} \in {\mathbb{C}}^{{2N_{T} N \times 2N_{R} N}}$ represents the block diagonal matrix composed of different subcarriers ${{\varvec{\Phi}}}(1)$, ${{\varvec{\Phi}}}(2)$, $\cdots$, ${{\varvec{\Phi}}}\left( N \right)$, which is related to the forward matrix ${\mathbf{A}}^{(1)}$ and ${\mathbf{H}}$, ${{\varvec{\Phi}}}\left( k \right) = {\mathbf{A}}^{(1)} \left( k \right)*{\mathbf{H}}\left( k \right)$. Separating the desired signal from the interfering signal in Eq. (14) could further lead to

15 $$ \begin{aligned} {\hat{\mathbf{d}}}^{1} & {\mathbf{ = A}}^{(1)} {\overline{\mathbf{Y}}} \\ & {\mathbf{ = \Phi d}} + {\mathbf{A}}^{(1)} {\mathbf{n}} \\ & {\mathbf{ = }}Diag\left( {{\varvec{\Phi}}} \right){\mathbf{d}} + \overline{Diag} \left( {{\varvec{\Phi}}} \right){\mathbf{d}} + {\mathbf{A}}^{(1)} {\mathbf{n}} \\ \end{aligned} $$

where $Diag( \cdot )$ denotes the diagonal matrix with diagonal elements. $\overline{Diag} ( \cdot )$ denotes the matrix with the remaining elements on the removed diagonal. The right side of Eq. (15) is composed of three parts. The first part is expected to obtain the signal, the second part can be assessed as the interference of the original signal, which can be defined as inter-antenna interference, and the third part is the noise interference of the original signal, which is mainly Gaussian noise interference. The second and third parts are considered as the interference of the original signal. The two parts are independent of the original signal. The i-th judgment variable is expressed as:

16 $$ \hat{d}_{i}^{1} {\mathbf{ = }}\left\{ {Diag\left( {{\varvec{\Phi}}} \right)} \right\}_{i,i} d_{i} + \left\{ {\overline{Diag} \left( {{\varvec{\Phi}}} \right){\mathbf{d}}} \right\}_{i} + \left\{ {{\mathbf{A}}^{(1)} {\mathbf{n}}} \right\}_{i} $$

where $\left\{ {\overline{Diag} \left( {{\varvec{\Phi}}} \right){\mathbf{d}}} \right\}_{i}$ denotes the i-th element of the orientation quantity $\overline{Diag} \left( {{\varvec{\Phi}}} \right){\mathbf{d}}$, the Gaussian interference noise term can be interpreted in the same way. At this point, the expected signal complex coefficient is denoted by $\varepsilon_{i} = \left\{ {Diag\left( {{\varvec{\Phi}}} \right)} \right\}_{i,i}$. The normalized expected signal can be further expressed by

17 $$ \overset{\lower0.5em\hbox{$\smash{\scriptscriptstyle\smile}$}}{d}_{i}^{1} {\mathbf{ = }}\hat{d}_{i}^{1} /\varepsilon_{i} = d_{i} + \left\{ {\overline{Diag} \left( {{\varvec{\Phi}}} \right){\mathbf{d}}} \right\}_{i} /\varepsilon_{i} + \left\{ {{\mathbf{A}}^{(1)} {\mathbf{n}}} \right\}_{i} \varepsilon_{i} $$

The use of soft detection allows for improved detection accuracy. When the likelihood value of the desired signal is computed, it is necessary to calculate the power of the second part of the interference term as well as the third part of the noise term employing Eq. (17). If the power of the interference part as well as the noise part need to be calculated, a priority must be given to calculating the power of the complex coefficients $\varepsilon_{i}$, and the average power $\varepsilon_{i}$ can be expressed by

18 $$ m_{s} = \left[ {\frac{{\sigma_{n}^{2} }}{{NN_{T} }}\sum\limits_{q = 1}^{{2NN_{T} }} {\left( {{\varvec{\Phi}}} \right)_{q,q} } } \right]^{2} $$

When the second part of the interference term is calculated, the power of each element of ${\mathbf{d}}$ is 1. The emitted signal is normalized by the power. Therefore, only the power of the $\overline{Diag} \left( {{\varvec{\Phi}}} \right)$ numerator term is required for the calculation. $\overline{Diag} \left( {{\varvec{\Phi}}} \right)$ denotes the i-th row of ${{\varvec{\Phi}}}$ with the elements on the diagonal removed from the remaining matrix. So, when the power of the second term $m_{I}^{i}$ is calculated, Eq. (19) is employed as follows:

19 $$ m_{I}^{i} = \left( {{\mathbf{\Phi \Phi }}^{H} } \right)_{i,i} - \left[ {\left( {{\varvec{\Phi}}} \right)_{i,i} } \right]^{2} $$

According to the average power of $\varepsilon_{i}$, the power of the second part can be further written as

20 $$ m_{I} = \left[ {\frac{{\sigma_{n}^{2} }}{{2NN_{T} }}\sum\limits_{q = 1}^{{2NN_{T} }} {\left( {{\mathbf{\Phi \Phi }}^{H} } \right)_{q,q} } } \right] - m_{s} $$

According to the same principle, the average power $m_{n}$ of the Gaussian interference noise in the third part is defined by

21 $$ m_{n} = \frac{{\sigma^{2} }}{{2NN_{T} }}\sum\limits_{q = 1}^{{2NN_{T} }} {\left( {{\mathbf{A}}^{(1)} \left( {{\mathbf{A}}^{(1)} } \right)^{H} } \right)}_{q,q} $$

After calculating the average power of $\varepsilon_{i}$, the total estimated signal power $m_{d}$ can be obtained as

22 $$ m_{d} = \left( {m_{I} + m_{n} } \right)/m_{s} $$

So, the estimated signal obeys a complex Gaussian random variable with zero mean and variance of $m_{d}$. The soft information likelihood score of the output of the first iteration is calculated based on the mean and variance to provide interference reconstruction a priori information for the second iteration. When the MAP detector output posterior LLR is computed to obtain a posteriori LLR function, we must opt for j corresponding code bit of i transmitted symbol, denoted as $d_{i}^{j}$.

23 $$ \begin{aligned} L\left( {d_{i}^{j} \left| {\hat{d}_{i} } \right.} \right) & = \ln \frac{{P\left( {d_{i}^{j} = 0\left| {\hat{d}_{i} } \right.} \right)}}{{P\left( {c_{i}^{j} = 1\left| {\hat{d}_{i} } \right.} \right)}} \\ & = \ln \frac{{\sum\nolimits_{{d \in \chi_{0}^{j} }} {P\left( {d_{i} = d\left| {\hat{d}_{i} } \right.} \right)} }}{{\sum\nolimits_{{d \in \chi_{1}^{j} }} {P\left( {d_{i} = d\left| {\hat{d}_{i} } \right.} \right)} }} \\ & { = }\ln \frac{{\sum\nolimits_{{d \in \chi_{0}^{j} }} {P\left( {\hat{d}_{i} \left| {d_{i} } \right. = d} \right)} }}{{\sum\nolimits_{{d \in \chi_{1}^{j} }} {P\left( {\hat{d}_{i} \left| {d_{i} } \right. = d} \right)} }} \\ \end{aligned} $$

where $\left[ {d_{i}^{0} ,d_{i}^{1} ,d_{i}^{2} , \cdots ,d_{i}^{M - 1} } \right]$ denotes the coded bit sequence corresponding to $d_{i}$. Two subsets of $\chi_{0}^{j} = \left\{ {\left. d \right|d^{j} = 0} \right\}$ and $\chi_{1}^{j} = \left\{ {\left. d \right|d^{j} = 1} \right\}$ represent the transmitted symbol subsets with the jth bit being 1 and 0, respectively. Using Eq. (24) $P\left( {d_{i} = d\left| {\hat{d}_{i} } \right.} \right)$ can be calculated as

24 $$ \begin{aligned} P\left( {d_{i} = d\left| {\hat{d}_{i} } \right.} \right) & = \prod\limits_{j = 0}^{{\log_{2} \left| \chi \right| - 1}} {P\left( {\left. {d_{i}^{j} = d^{j} } \right|\hat{d}_{i} } \right)} \\ & = C\prod\limits_{j = 0}^{{\log_{2} \left| \chi \right| - 1}} {\exp \left( { - d_{i}^{j} L\left( {d_{i}^{j} } \right)/2} \right)} \, \\ \end{aligned} $$

where $C$ is a constant, and $C^{ - 1} = \prod\limits_{j = 0}^{{\log_{2} \left| \chi \right| - 1}} {\exp \left( {L\left( {d_{i}^{j} } \right)/2} \right) + \exp \left( { - L\left( {d_{i}^{j} } \right)/2} \right)}$, $\left| \chi \right|$ indicates the size of the symbol set $\chi$. The conditional variance in Eq. (21) is employed and a new expression to calculate the LLR is attained as follows:

25 $$ L\left( {{\text{c}}_{i}^{j} } \right) = \ln \frac{{\sum\limits_{{d \in \chi_{0}^{j} }} {\exp \left( { - \frac{{\left| {\hat{d}_{i} - d} \right|^{2} }}{{\upsilon_{{\hat{d}_{i} }} }}} \right)} }}{{\sum\limits_{{d \in \chi_{1}^{j} }} {\exp \left( { - \frac{{\left| {\hat{d}_{i} - d} \right|^{2} }}{{\upsilon_{{\hat{d}_{i} }} }}} \right)} }} $$

So far, the LLRs can be obtained by calculating Eqs. (23) through (25). The LLRs are finally transferred to the soft decoder by Eq. (25).

b. After the first iteration ($m \ge 2$)

During the second and subsequent iterations, Feedback currently exists. Therefore, the feedback and the normalization matrices have different forms when compared to the first iteration. For simplicity of the explanation, the use of matrix ${\mathbf{M}}\left( k \right)$ is introduced before instead of employing ${\mathbf{H}}_{{{\text{equ}}}} \left( k \right){\mathbf{T}}\left( k \right)^{ - 1} {\mathbf{P}}\left( k \right)^{ - 1}$, i.e., ${\mathbf{M}}\left( k \right) = {\mathbf{H}}_{{{\text{equ}}}} \left( k \right){\mathbf{T}}\left( k \right)^{ - 1} {\mathbf{P}}\left( k \right)^{ - 1}$. For a more convenient description of the algorithm, giving priority to the case of a single subcarrier, the forward matrix can then be a channel-matching matrix defined by

26 $$ {\mathbf{A}}^{(m)} \left( k \right) = {\mathbf{M}}^{H} \left( k \right) $$

where $k$ denotes the subcarrier sequence, which is the same as in Eq. (11). The second and subsequent forward matrices have ${\mathbf{A}}^{(m)} \in {\mathbb{C}}^{{2N_{T} N \times 2N_{R} N}}$ for all subcarriers, which is a block diagonal matrix composed of ${\mathbf{A}}^{(m)} \left( 1 \right),{\mathbf{A}}^{(m)} \left( 2 \right), \cdots ,{\mathbf{A}}^{(m)} \left( N \right)$. After the received data symbols are processed by the forward matrix defined by

27 $$ {\mathbf{A}}^{(m)} \left( k \right){\overline{\mathbf{Y}}}\left( k \right){\mathbf{ = M}}^{H} \left( k \right){\mathbf{M}}\left( k \right){\mathbf{d}}\left( k \right) + {\mathbf{M}}^{H} \left( k \right){\mathbf{n}}\left( k \right) $$

Thus, the interference of the desired signal mainly comes from the elements on the ${\mathbf{H}}_{{{\text{equ}}}}^{H} \left( k \right){\mathbf{H}}\left( k \right)$ non-diagonal in Eq. (27). If the ${\mathbf{H}}_{{{\text{equ}}}}^{H} \left( k \right){\mathbf{H}}\left( k \right)$ non-diagonal elements can be eliminated after the power normalization process is run, the desired signal is obtained. Following this idea, through the last judgment feedback signal ${\hat{\mathbf{d}}}^{(m - 1)} \left( k \right)$ using reconstruction interference, the feedback matrix ${\mathbf{B}}^{(m)} \left( k \right)$ is defined by

28 $$ {\mathbf{B}}^{(m)} \left( k \right) = {\mathbf{M}}^{H} \left( k \right){\mathbf{M}}\left( k \right) - \, diag\left( {{\mathbf{M}}^{H} \left( k \right){\mathbf{M}}\left( k \right)} \right) $$

Then, the normalization matrix ${\mathbf{W}}^{(m)} \left( k \right)$ after interference cancellation is defined by

29 $$ {\mathbf{W}}^{(m)} \left( k \right) = \left\{ {diag{(}{\mathbf{M}}^{H} \left( k \right){\mathbf{M}}\left( k \right)} \right\}^{ - 1} $$

For all carriers, the feedback and normalization matrices,${\mathbf{B}}^{(m)} \in {\mathbb{C}}^{{2N_{T} N \times 2N_{R} N}}$ and ${\mathbf{W}}^{(m)} \in {\mathbb{C}}^{{2N_{T} N \times 2N_{R} N}}$ , are block diagonal matrices consisting of ${\mathbf{B}}^{(m)} (1),{\mathbf{B}}^{(m)} (2), \cdots ,{\mathbf{B}}^{(m)} (N)$ and ${\mathbf{W}}^{(m)} (1),{\mathbf{W}}^{(m)} (2), \cdots ,{\mathbf{W}}^{(m)} (N)$, respectively. The main difference between the second and subsequent iterative methods when compared to the first iteration is the presence of feedback information during the second and subsequent iterations, which requires interference reconstruction employing the soft information from the feedback of the previous iteration. The soft information output from the previous iteration is defined as ${{\varvec{\uplambda}}}^{(m - 1)}$, and the interference cancellation is performed by reconstructing the signal vector ${{\varvec{\upmu}}} = \left[ {\mu (1),\mu (2), \cdots ,\mu \left( N \right)} \right]^{T}$ by using Eq. (25). The reconstructed inter-antenna interference is obtained by multiplying the reconstructed interference signal with the feedback matrix ${\mathbf{B}}^{(m)}$.

According to the forward, feedback, and normalization matrices in Eqs. (27) through (29) and the reconstructed signal vector ${{\varvec{\upmu}}}$, the judgment vector of $m \ge 2$ iterations can be obtained as

30 $$ \begin{aligned} {\hat{\mathbf{d}}}^{(m)} & = {\mathbf{W}}^{(m)} [{\mathbf{A}}^{(m)} {\mathbf{Y}} - {\mathbf{B}}^{(m)} {{\varvec{\upmu}}}] \\ & = {\mathbf{W}}^{(m)} \left\{ {\left[ {\left( {{\mathbf{W}}^{(m)} } \right)^{ - 1} + {\mathbf{B}}^{(m)} } \right]{\mathbf{d}} + {\mathbf{A}}^{(m)} {\mathbf{n}} - {\mathbf{B}}^{(m)} {{\varvec{\upmu}}}} \right\} \\ & = {\mathbf{d}} + {\mathbf{W}}^{(m)} {\mathbf{B}}^{(m)} ({\mathbf{d}} - {{\varvec{\upmu}}}) + {\mathbf{W}}^{(m)} {\mathbf{A}}^{(m)} {\mathbf{n}} \\ \end{aligned} $$

The right side of Eq. (30) is composed of three parts. The first part is the original signal, the second part can be considered as the error appearing in the reconstructed signal, which is also defined as the error interference and the third part is the noise interference of the original signal.

The second as well as the later iterations are based on soft information. The likelihood score of ${\hat{\mathbf{d}}}^{(m)}$ regarding the second and later iterations of the detector output is obtained. The power of the second and third parts in Eq. (30) is required. Therefore, the error interference power is defined as $m_{e}$ and the noise power as $m_{n}$, respectively. For the error interference part, ${\mathbf{e}} = {\mathbf{d}} - {{\varvec{\upmu}}}$ is defined, then the interference of the $i$-th element of the estimated signal is obtained as the product of the $i$-th row of the coefficient matrix ${\mathbf{W}}^{(m)} {\mathbf{B}}^{(m)}$ and the error vector ${\mathbf{e}}$. Then, the power of the real part of the $i$ th element in the error vector is represented by

31 $$ \begin{aligned} E\left[ {Re\left( {e_{i} } \right)^{2} \left| {\hat{d}_{i}^{(m)} } \right.} \right] & = E\left[ {Re\left( {d_{i} - \mu_{i} } \right)^{2} } \right] \\ & = 1 - {\text{tanh}}^{{2}} \left( {\lambda_{i}^{I} } \right) \\ \end{aligned} $$

In^25^, the coefficient matrix is an upper triangular matrix composed of 0s on the diagonal, the power of the coefficient matrix in the calculation of the second part of Eq. (30) can be derived as follows:

32 $$ \begin{aligned} P^{m} & = {\text{var}} \left[ {\left( {{\mathbf{W}}^{(m)} {\mathbf{B}}^{(m)} } \right)_{p,q} } \right] \\ & { = }\frac{1}{{\left( {2NN_{T} } \right)^{2} }}\sum\limits_{p = 1}^{{2NN_{T} }} {\sum\limits_{q = 1}^{{2NN_{T} }} {\left| {\left[ {{\mathbf{W}}^{(m)} {\mathbf{B}}^{(m)} } \right]_{p,q} } \right|} }^{2} \\ \end{aligned} $$

The power of the error interference is defined by

33 $$ m_{e} = P^{m} \sum\limits_{i = 1}^{N} {E\left[ {Re\left( {e_{i} } \right)^{2} \left| {\hat{d}_{i}^{(m)} } \right.} \right]} $$

According to the same principle of the first calculation of $m_{n}$,$m_{n}$ are represented by

34 $$ \begin{aligned} m_{n} & = \frac{1}{{2NN_{T} }}\sum\limits_{q = 1}^{{2NN_{T} }} {\left( {{\mathbf{W}}^{(m)} {\mathbf{R}}_{n} {\mathbf{W}}^{(m)H} } \right)}_{q,q} \\ & = \frac{{\sigma_{n}^{2} }}{{2NN_{T} }}\sum\limits_{q = 1}^{{2NN_{T} }} {\left( {{\mathbf{W}}^{(m)} {\mathbf{W}}^{(m)H} } \right)}_{q,q} \\ \end{aligned} $$

The power of the estimated total signal $m_{d}$ is denoted by $m_{d} = m_{e} + m_{n}$. Finally, the likelihood score of the current iteration is calculated. It is worth noting that the output likelihood score is the sum of the current likelihood score and the output likelihood score of the previous iteration, i.e., $\lambda^{m} = \lambda + \lambda^{m - 1}$ due to the influence of the signal error term at this point. Finally, the desired signal could be obtained by adjudicating the likelihood score.

The pseudocode for the algorithm is presented below:

**Algorithm 1:**
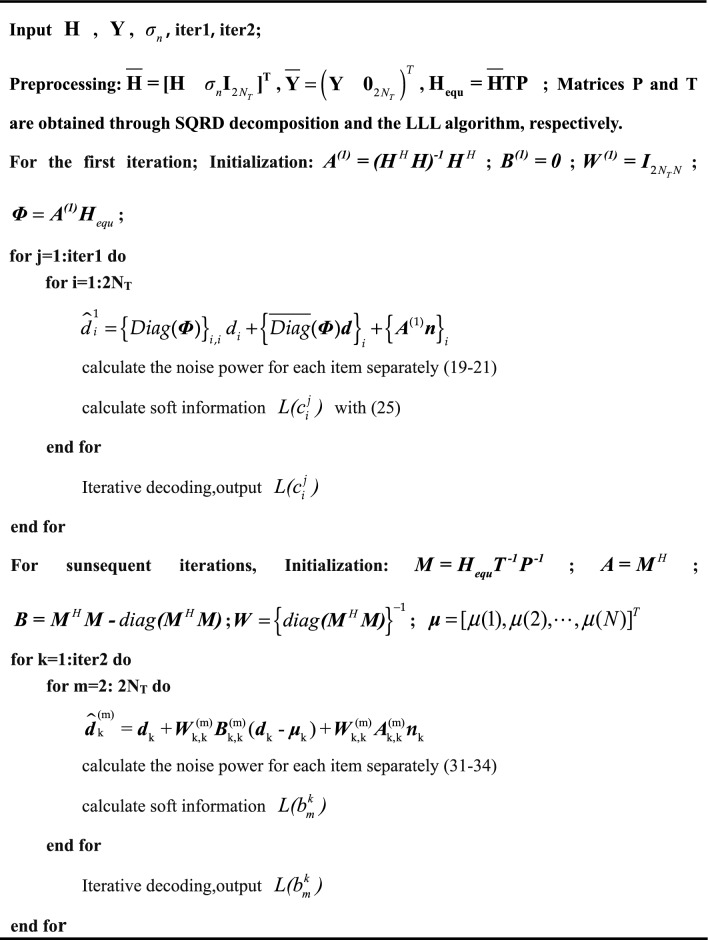
Proposed IPIC LRA algorithm

To better present the research outcomes in the paper, we find it imperative to elaborate on the introduction of the IPIC-LRA method. Using the extraction of the kth carrier as an instance, the algorithm's specific steps are summarized as follows:

**S1** First initialize and also set the maximum number of iterations. After that, the LLL algorithm is utilized and the channel matrix ${\mathbf{H}}_{{{\text{equ}}}}$ and ${\mathbf{P}}$ and ${\mathbf{T}}$ matrices are reconstructed.

**S2** Using Eqs. (10) through (13), calculate the forward, the power normalization, and the feedback matrices as ${\mathbf{A}}^{(1)} = \left( {{\mathbf{H}}_{{{\text{equ}}}}^{H} \left( k \right){\mathbf{H}}_{{{\text{equ}}}} \left( k \right)} \right)^{ - 1} {\mathbf{H}}_{{{\text{equ}}}}^{H} \left( k \right)$,${\mathbf{W}}^{(1)} = {\mathbf{I}}_{{2N_{T} N}}$,${\mathbf{B}}^{(1)} = 0$, respectively. The estimated signal ${\hat{\mathbf{d}}}^{1}$ will be obtained using Eq. 14. According to Eqs. (18) through (22), the total power $m_{d}$, the $m_{s}$,$m_{n}$, and $m_{I}$ can be calculated separately. The likelihood score of the estimated signal ${\hat{\mathbf{d}}}^{1}$ is calculated. After that, the next iteration is executed.

**S3** Recalculate the forward, feedback, and power normalization matrices as ${\mathbf{A}}^{(m)} = {\mathbf{M}}^{H}$, ${\mathbf{B}}^{(m)} = {\mathbf{M}}^{H} {\mathbf{M}} - \, diag\left( {{\mathbf{M}}^{H} {\mathbf{M}}} \right)$, ${\mathbf{W}}^{(m)} = \left\{ {diag\left( {{\mathbf{M}}^{H} {\mathbf{M}}} \right)} \right\}^{ - 1}$, respectively.

**S4** Using Eq. (30), the current estimated signal ${\hat{\mathbf{d}}}^{(m)}$ is obtained. According to Eqs. (32) through (34), the different powers will be calculated separately. Finally, the likelihood score of the estimated signal ${\hat{\mathbf{d}}}^{(m)}$ is calculated. After that, the next iteration is performed. Continue with **S4** if it is not greater than the maximum number of iterations. It is worth noting that the output likelihood score is the sum of the current likelihood score and the output likelihood score of the previous iteration at this time. If the maximum number of iterations is exceeded, then proceed to **S5.**

**S5** The estimated signal likelihood score is calculated and transferred to the turbo decoder.

## Complexity

In^26^, the complexity of the conventional matrix operations is summarized as follows:

The complexity of multiplying ${\mathbf{Ab}}$ by a $m \times m$ dimensional matrix ${\mathbf{A}}$ and the vector ${\mathbf{b}}$ is $m(2m - 1)$. The complexity of adding a $m \times m$ dimensional matrix ${\mathbf{A}}$ with ${\mathbf{B}}$ is $m^{2}$. The complexity of inverting a $m \times m$ dimensional matrix ${\mathbf{A}}$ is $2m^{3} - 2m^{2} + m$.

The linear detector based on the MMSE is employed in the first detection. Considering that the channel matrix is diagonal, in the process of calculating the judgment variables, the complexity of the matrix, the multiplication and addition operations between the vectors are $\left( {N_{R} + 3} \right)N^{2}$, while the operation of matrix inversion is $2N^{3} - 2N^{2} + N$. The sum of the two operations has the complexity of linear detection before the soft judgment is performed. The iterative detection is the first and later processes are different. The complexity of the first iteration is considered first. In the process of calculating the complex coefficients $\varepsilon_{i}$ of the desired signal, as well as the interference and Gaussian noise power, it is necessary to use the matrix ${{\varvec{\Phi}}}$ of the form ${{\varvec{\Phi}}} = {\mathbf{A}}^{(1)} {\mathbf{H}}$. At this point, the increased complexity is the product of the matrices ${\mathbf{A}}^{(1)}$ and ${\mathbf{H}}$. The reduction is the complexity of the ${\mathbf{H}}^{H} {\text{y}}$ part. Therefore, the complexity of calculating ${{\varvec{\Phi}}}$ is $2\left( {N_{R} - 2} \right)N^{2} + N$. The complexity of calculating the complex coefficients, as well as the complexity of the interference and Gaussian noise power, can be obtained from the matrix ${{\varvec{\Phi}}}$. To obtain $\varepsilon_{i} = \left( {{\varvec{\Phi}}} \right)_{i,i}$, the complexity of $\varepsilon_{i}$ is $4N^{2} \log_{2} N$. To calculate the disturbance power, the matrix product ${\mathbf{\Phi \Phi }}^{H}$ introduces a large complexity when finding the first disturbance $m_{I}$. So, the complexity of the total operation is $4N^{3} - 2N^{2} + 2N$, and the complexity of $m_{s}$ is $2N + 2$. When calculating the Gaussian noise $m_{n}$, which also involves the matrix product, the complexity introduced is $8N^{3} - 4N^{2} + 2N{ + }2$, and the complexity of the final calculation of the likelihood score is $2N$.

In the introduced LLL algorithm, the added complexity is the number of column exchanges of the base matrix $N^{2} \log_{2} B$, where B is the longest base vector norm, so the total complexity of lattice reduction is $N^{4} \log_{2} B$. In the actual detection, the complex matrix is transformed into an equivalent real matrix, but this will double the channel matrix and the complexity will become $2N^{4} \log_{2} B$. The total complexity of the first iteration detection is $2N^{4} \log_{2} B{ + }14N^{3} - \left( {3N_{R} + 9} \right)N^{2} + 4N^{2} \log_{2} N) + 8N$.

In the second and subsequent iterations, the complexity decreases significantly for the first iteration. ${\mathbf{M}}$, ${\mathbf{B}}^{(m)} = {\mathbf{M}}^{H} {\mathbf{M}} - \, diag\left( {{\mathbf{M}}^{H} {\mathbf{M}}} \right)$ and ${\mathbf{W}}^{(m)} = \left\{ {diag\left( {{\mathbf{M}}^{H} {\mathbf{M}}} \right)} \right\}^{ - 1}$ is already calculated in the first iteration. The increase in complexity is only $4N$. When calculating the residual interference power, the complexity is mainly introduced by two parts. The operation to calculate the $m_{e}$ is $4N$, and the other part is the $P^{m}$. The matrix ${\mathbf{W}}^{(m)}$ is diagonal, and the operation to calculate $P^{m}$ is $6N^{2}$. The complexity of the interference reconstruction is $4N^{2} + 2N\log_{2} N - 2N$. The complexity of power normalization and interference cancellation is $4N$. The total complexity of the second iteration is $10N^{2} + 2N\log_{2} N + 10N$.

Thus, the complexity of the IPIC-LRA increases significantly when MMSE is used in the first iteration. But, the complexity of the second detection iteration is ${\rm O}\left( {N^{2} } \right)$, which is significantly lower than that of the first iteration and linear detection, and the complexity of the later iterations is lower than that of the second iteration because there is no need to calculate $P^{m}$ again.

## The results of the simulation

Several algorithms are compared with the proposed algorithm by using the Monte Carlo simulation technique. The channel model is assumed to be a Rayleigh fading channel with ideal channel estimation. The system configuration is composed of a receiving user, system bandwidth with 10 MHz, the normal CP frame structure, the transmission time interval (TTI) per frame containing 14 OFDM symbols, and the bandwidth configured to 512 subcarriers, where 300 of which are used for data symbol transmission. The other carriers are set to dummy subcarriers with a frequency spacing of 15 kHz, modulation types of 4QAM, receiving and transmitting antenna configurations of 4 × 4 or 6 × 6, turbo code using the generated polynomials (7,5) or (11,13), coding rate of 1/2, and log-map decoding mode. The specific parameters are shown in Table 1.

**Table 1:** Experimental simulation parameters.

Silmulation parameters	Figures
Number of users	1
Channel	Multipath Rayleigh fading channel
System bandwidth	10 MHz
Carrier spacing	15 kHz
Number if inverse Fourier transform point	512
The number of transmitted data symbols	300
Number of data symbols	14
Modulation	4QAM
Number of transmitting and receiving antennas	4 × 4; 6 × 6
Channel coding	Turbo decoding mode
Channel decoding mode	Log-map

**Figure 2:**
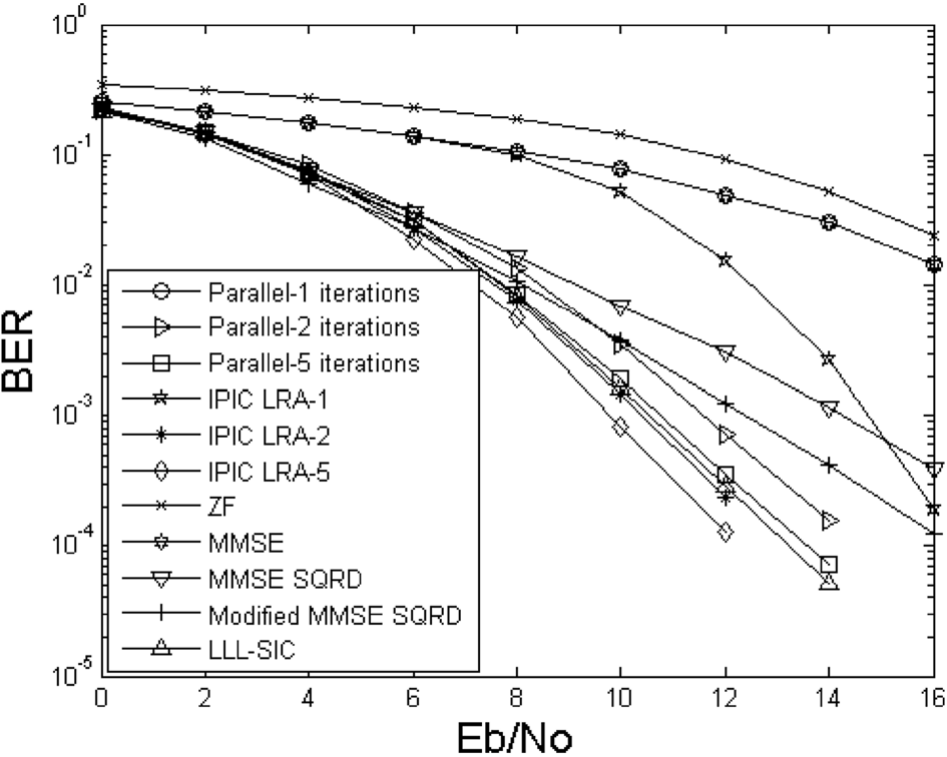
BER for demodulating the output of different methods using $N_{T} = N_{R} = 4$.

**Figure 3:**
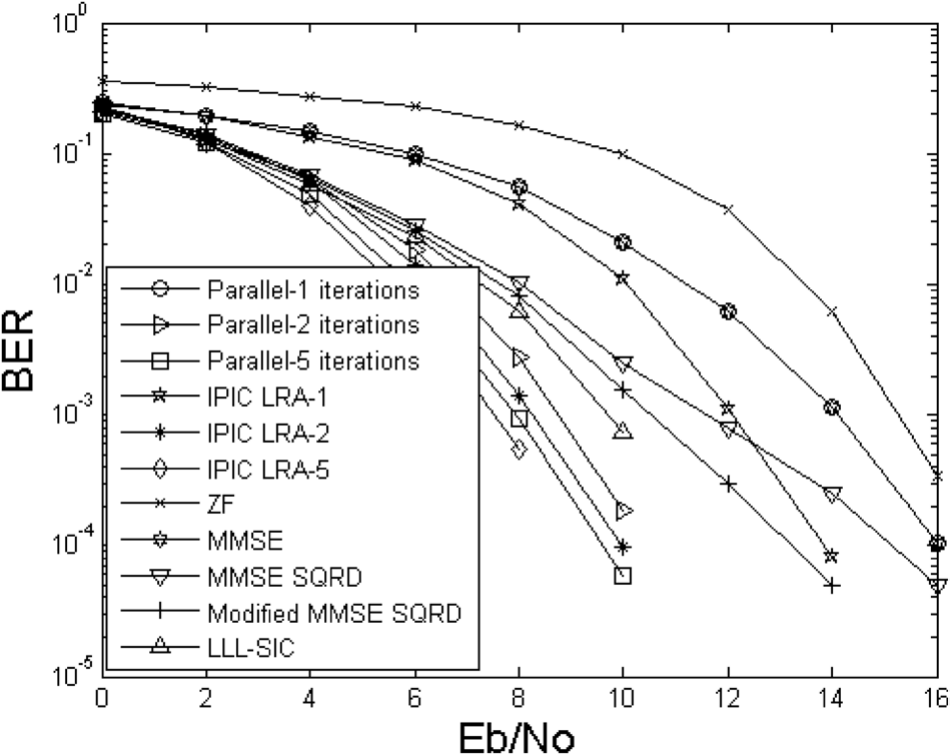
The BER for demodulating the output of different methods using $N_{T} = N_{R} = 6$.

Figures 2 and 3 illustrate the bit error rate comparison curves of the proposed IPIC-LRA algorithm with other algorithms. During the comparison, we kept the critical parameter AA of the LLL algorithm fixed at $\delta { = }0.75$, selected 4QAM as the modulation scheme, with antennas labeled as 4 × 4 and 6 × 6, respectively. The figures indicate that the algorithmic performance improvement of linear detection methods is relatively slow, irrespective of the antenna configuration. Nonlinear methods, including MMSE SQRD and Modified MMSE SQRD^16^, show a more pronounced enhancement compared to linear methods. When configuring antenna 6 × 6, the performance improvement of both MMSE SQRD and Modified MMSE SQRD algorithms is approximately 3 to 5 dB at a bit error rate of 10^–3^. Applying the LLL principle to the SIC algorithm reveals that the algorithm's performance can still be further improved. This is because the LLL principle can restore the orthogonality of the channel matrix, but compared to the PIC method, the performance improvement remains relatively slow. In Fig. 3, The PIC method is used based on the MMSE criterion, and its performance is slightly improved. In the first iteration, the IPIC-LRA algorithm introduces the LLL algorithm, which can effectively optimize the channel matrix and make it have more strict orthogonality, and the BER improvement is obvious. At BER of 10^–2^, the improvement of the IPIC-LRA is about 2.5 dB. As the number of iterations increases, the IPIC-LRA gradually converges and stabilizes. In the second iteration, the performance of the IPIC-LRA improves by about 0.8–1.5 dB when the BER is 10^–3^. After running five iterations, when the BER is 10^–4^, the performance of the IPIC-LRA is improved by about 0.8–2 dB when compared with the benchmark algorithm.

**Figure 4:**
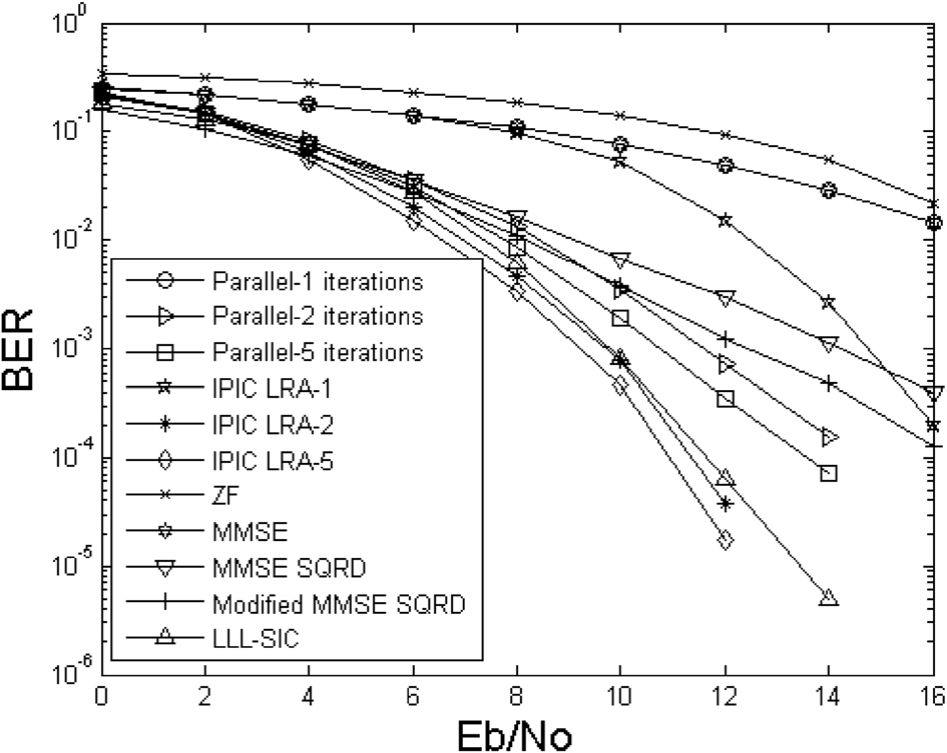
The BER for demodulating the output of different methods using $\delta { = }0.99$.

Figure 4 shows the BER of IPIC LRA and others under 4QAM modulation and the LLL ($\delta { = }0.99$) condition in an 4 × 4 arrangement. Compared to Fig. 3, both the IPIC LRA and LLL-SIC algorithms are affected by the introduction of $\delta$. As $\delta$ increases, the performance of these two methods can improve rapidly. At a bit error rate of 10^–4^, the IPIC LRA algorithm achieves an Eb/No of approximately 11 dB with $\delta$ at 0.99, and 12 dB with $\delta$ at 0.75.The findings reveal that the proposed IPIC-LRA method outperforms other approaches in terms of the BER, highlighting its potential for high-performance wireless communication systems. Besides, the performance improvement rate of the IPIC-LRA is still obvious when compared to other algorithms. In the first iteration, the performance of the proposed IPIC-LRA method is improved very rapidly when compared to the first iteration of the benchmark algorithm, where the BER is set to 10^–2^. So, the system performance improvement is already more than about 4 dB. As the number of iterations increases, when the BER is set to 10^–4^, the performance of the proposed IPIC-LRA is 1.8 to 2.2 dB, which is better than that of the parallel method when the number of iterations is set from the second iteration to five iterations.

**Figure 5:**
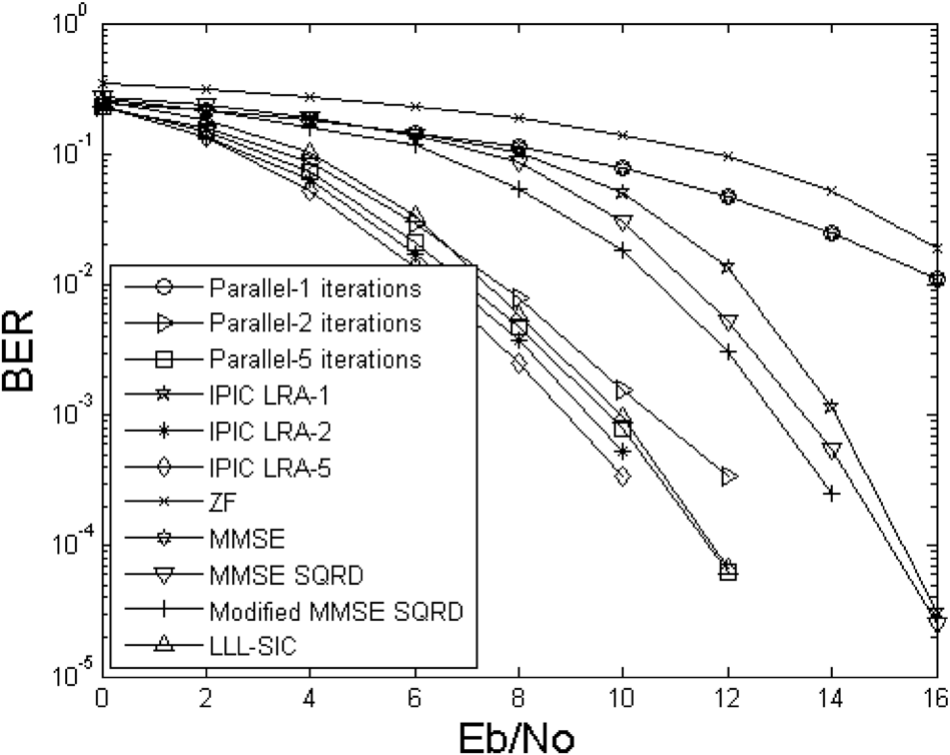
The BER of the demodulating output of different methods for the generated polynomial (11, 13).

The BER of different methods under the LLL (0.75) and the generated polynomial (11, 13) condition in the 4 × 4 arrangement are shown in Fig. 5.Considering the adoption of different polynomials with the aim of improving algorithm performance at the same error rate. Simultaneously, whether using linear or nonlinear detection methods, Fig. 5 exhibits a significant performance improvement compared to Fig. 3. The Turbo polynomial transitions from (7,5) to (11,13), enhancing performance by introducing additional coding redundancy, explaining the marked change in the performance curve.The proposed IPIC-LRA algorithm is about 0.8 dB more than that of the parallel algorithm in the initial iteration. At the second iteration, when the BER is set to 10^–3^, similar outcomes are reached. The number of iterations reaches 5 and the performance improvement of the IPIC-LRA algorithm becomes better when compared to the parallel algorithm.

## Conclusion

The present research introduces an iterative parallel interference cancellation receiver based on a lattice reduction-aided approach that utilizes the LRA to enhance the orthogonality between constellation points and the MMSE extension to mitigate the noise effects. The proposed method aims to improve the performance of wireless communication systems by increasing the accuracy of symbol detection while reducing the impact of interference.

The manuscript highlights the potential of the proposed algorithm to enhance the efficiency of communication systems by facilitating reliable information transmission. The proposed method is combined with the LLR clipping to decrease the list size necessary to achieve the highest achievable performance. The parallel detection algorithm depends heavily on the accuracy of the initial score of the detection. Therefore, the LRA idea is introduced into the algorithm during the initial detection in the article.

The simulation results make the initial detection effectively very obvious and far better than other algorithms after the correction of the LRA idea. It implies that as the number of iterations increases, the performance of the proposed algorithm would grow significantly when compared with other algorithms.

## Data Availability

The code written and analyzed during the current study are not publicly available due to the confidentiality but are available from the corresponding author on reasonable request.
